# Preterm Birth Prevention Post-Conization: A Model of Cervical Length Screening with Targeted Cerclage

**DOI:** 10.1371/journal.pone.0163793

**Published:** 2016-11-03

**Authors:** Lindsay M. Kindinger, Maria Kyrgiou, David A. MacIntyre, Stefano Cacciatore, Angela Yulia, Joanna Cook, Vasso Terzidou, T. G. Teoh, Phillip R. Bennett

**Affiliations:** 1 Institute of Reproductive and Developmental Biology, Department of Surgery and Cancer, Imperial College, London, United Kingdom; 2 Department of Obstetrics and Gynaecology, St Mary’s Hospital, Imperial College Healthcare NHS Trust, London, United Kingdom; 3 Department of Obstetrics and Gynaecology, Queen Charlotte’s and Chelsea—Hammersmith Hospital, Imperial College Healthcare NHS Trust, London, United Kingdom; 4 Department of Obstetrics and Gynaecology, Chelsea and Westminster Hospital NHS Trust, London, United Kingdom; Fetal Medicine and Surgery, MEXICO

## Abstract

Women with a history of excisional treatment (conization) for cervical intra-epithelial neoplasia (CIN) are at increased risk of preterm birth, perinatal morbidity and mortality in subsequent pregnancy. We aimed to develop a screening model to effectively differentiate pregnancies post-conization into low- and high-risk for preterm birth, and to evaluate the impact of suture material on the efficacy of ultrasound indicated cervical cerclage. We analysed longitudinal cervical length (CL) data from 725 pregnant women post-conization attending preterm surveillance clinics at three London university Hospitals over a ten year period (2004–2014). Rates of preterm birth <37 weeks after targeted cerclage for CL<25mm were compared with local and national background rates and expected rates for this cohort. Rates for cerclage using monofilament or braided suture material were also compared. Of 725 women post-conization 13.5% (98/725) received an ultrasound indicated cerclage and 9.7% (70/725) delivered prematurely, <37weeks; 24.5% (24/98) of these despite insertion of cerclage. The preterm birth rate was lower for those that had monofilament (9/60, 15%) versus braided (15/38, 40%) cerclage (RR 0.7, 95% CI 0.54 to 0.94, *P* = 0.008). Accuracy parameters of interval reduction in CL between longitudinal second trimester screenings were calculated to identify women at low risk of preterm birth, who could safely discontinue surveillance. A reduction of CL <10% between screening timepoints predicts term birth, >37weeks. Our triage model enables timely discharge of low risk women, eliminating 36% of unnecessary follow-up CL scans. We demonstrate that preterm birth in women post-conization may be reduced by targeted cervical cerclage. Cerclage efficacy is however suture material-dependant: monofilament is preferable to braided suture. The introduction of triage prediction models has the potential to reduce the number of unnecessary CL scan for women at low risk of preterm birth.

## Introduction

Women with a history of excisional treatment (conization) for cervical intra-epithelial neoplasia (CIN) have a shorter cervical length (CL) in pregnancy than those without treatment [1], significantly increasing their risk of preterm birth <37weeks, perinatal morbidity and mortality [2–8]. Although the underlying mechanisms are unclear, hypotheses include immunomodulation relating to HPV infection affecting biochemical pathways to parturition, and ‘mechanical weakness’ secondary to loss of cervical tissue [9]. Second trimester CL measurements in pregnancy post-conization are as predictive for preterm birth [1, 10, 11] as they are for the general obstetric population, in whom a cut-off of 25 mm is widely used as a treatment threshold [10, 12–14]. Pregnancies post-conization account for an increasing proportion of referrals to preterm CL surveillance clinics; from none in 1999, to more than 40% of referrals in 2012 [15]. The antenatal management of those found to have a short cervix is largely unstandardized and remains clinician and unit-dependant. Evidence for progesterone treatment is lacking, and while a cerclage is often inserted to mechanically support the deficient cervix [16], increasing evidence from retrospective case series suggest that cerclage insertion is either of no benefit, or may even worsen outcome through increased preterm birth rates [17–20]. However these were studies in cohorts in whom cerclage was performed using braided suture material; a reflection of current global clinical practice. In the UK Mersilene™, a non-absorbable braided polyester suture, is used by over 80% of obstetricians in preference to a monofilament alternative such as Nylon or Prolene [21], despite a lack of evidence-base. The only study to report on the effect of suture material on cerclage efficacy compared two types of braided suture, Ethibond™ and Mersilene™, and excluded all monofilament cerclages. This study showed no difference in preterm birth rates [22].

The multifilament structure of braided suture favours bacterial colonisation over monofilament suture [23–25], and is associated with poorer wound healing [26–28]. This may explain the doubled risk of puerperal pyrexia associated with cerclage insertion [29], and the lack of any observed benefit of a cerclage for preterm birth prevention post-conization [17–20]. Hypothesizing that suture material effects cerclage efficacy post-conization, we compared rates of preterm birth for monofilament versus braided suture material in a retrospective observational study of cerclage procedures across three hospitals, over ten years.

Despite accounting for a large proportion of referrals for labour intensive and costly ultrasound-directed preterm surveillance, the majority of pregnancies post-conization will deliver at term (80–86%) without intervention [2, 5]. Therefore a secondary aim of this study to develop a triage prediction model to clearly differentiate high-risk women, in whom targeted intervention may be beneficial, from those at low-risk of preterm birth who could discontinue surveillance in a safe and timely manner.

## Methods

A retrospective observational study was conducted in the preterm surveillance clinics at three London University maternity units (Queen Charlottes Hospital, St Marys Hospital, Chelsea Westminster Hospital) from January 2004 to January 2014. We included all women during their first singleton pregnancy after excisional cervical treatment for CIN of depth ≥12mm [30] (including Cone biopsy, LLETZ and LEEP). We included women that required an ultrasound-indicated cerclage, but excluded those undergoing a pre-planned or history-indicated cervical cerclage. Women were only eligible if they had no other risk factors for preterm birth; any women with a prior preterm delivery (<37 weeks), mid-trimester miscarriage (>13 weeks), uterine anomaly or a multi-fetal pregnancy in the index pregnancy preterm were excluded. Women were included for analysis if CL measurements were available for at least one of three screening time-points; A: 13^+0^–15^+6^ weeks, B: 16^+0^–18^+6^ weeks, and C: 20^+0^–22^+6^ weeks. This was retrospective analysis of previously collected, anonymized data and did not require ethics approval.

All hospitals implemented a pre-specified surveillance protocol, applicable to all women attending prematurity clinics across sites for the duration of the study. This included serial mid-trimester CL measurements and cervical cerclage for those with a cervix shorter than 25mm before 24 weeks of gestation. CL measurements were taken at trans-vaginal ultrasound (TVUS) with an empty bladder, avoiding undue pressure on the cervix, and fundal pressure applied to illicit any further cervical shortening.

The primary objectives were to assess the overall efficacy of cerclage in women with a shortened cervix post-conization, and the effect of suture material on preterm birth rates. The overall preterm birth rates in the observed cerclage study population were therefore compared to background preterm birth rates in the general obstetric population at the three university hospitals, and to national preterm birth rates for England and Wales as taken from The Office of National Statistics Data UK 2005 (mid-point in the study period). Fisher’s exact test was used to assess whether the rate of preterm birth <37 weeks differs in women with a monofilament versus braided cerclage.

The second objective was to develop a triage prediction model to differentiate women at low-risk for preterm birth in whom CL surveillance is unnecessary, from women at high-risk. The study cohort was classified into two groups. Group 1 were ‘low-risk’; they delivered at term, did not demonstrate cervical shortening and did not receive cerclage intervention. Group 2 were considered ‘high-risk’ as they either had an ultrasound-indicated cerclage for cervical shortening below 25mm, or delivered prematurely (less than 37 weeks of gestation).

To determine if these pre-defined high and low risk groups demonstrated differing patterns of cervical shortening at screening, Group 1 and Group 2 were assessed for the percentage change in CL (%ΔCL) between serial screening time-points (A, B and C), using Kruskal-Wallis and Mann Whitney tests. Only CL measurements made before the insertion of cerclage were included in the analysis.

We calculated the accuracy of different thresholds for percentage ΔCL (≥5%, ≥10%, ≥20%, ≥30%, ≥40%) in predicting preterm birth (<37 weeks) and/or the need for cerclage, as well as the accuracy of single CL measurements (≤15mm, ≤20mm, ≤25mm, ≤30mm and ≤35mm). Accuracy parameters included sensitivity (S), specificity (Sp), positive (PPV), negative predictive value (NPV) and likelihood ratios (LR). Receiver operator curves (ROC) were plotted and used to determine optimum CL thresholds.

A triage prediction model was developed using decision tree analysis (R package “rpart”[31]) to identify pregnancies at low-risk of preterm birth that could safely discontinue serial surveillance. The model was designed to predict term birth (beyond 36^+6^ weeks) without requirement for cerclage with high sensitivity and a low false positive rate (5%), ensuring a minimal number of women that delivered preterm were falsely classified as ‘low-risk’ within the model. These highly conservative margins ensured identification of only the lowest risk women suitable for discharge from surveillance. To assess the prediction ability, the model was built on two independent sets and tested on the remaining set. The whole procedure was repeated three times testing each hospital location. The model consists of 3 steps. In the first step, CL at timepoint A was used as predictor. Two thresholds were defined to classify the participant as high risk, low risk, or requiring further screening at timepoint B and/or C. In the second step, the CL at time-point B and change in CL between A and B were used as predictors. In the third step, the classification tree was used as final predictor using as variables CL at time-point A, time-point B and time-point C, and all the variations between timepoints.

The produced algorithm was then applied retrospectively to our cohort to calculate the number of unnecessary ultrasound scans. Based on an assumption that each patient attended for at least three transvaginal scans, we estimated the total number of transvaginal scans that could have been prevented.

Potential sources of bias were considered. Notably this included selection of participants, as only data on women referred to prematurity clinics were included. Selection of suture material for cerclage may have been biased as the retrospective design precluded randomization.

## Results

A total of 725 pregnant women post-conization were included in the analysis. The patient characteristics and the distribution into low-risk term birth (Group 1) and those delivering preterm or receiving an ultrasound indicated cerclage (Group 2) are described in Table 1. Age, BMI, and ethnicity were comparable amongst the two groups. Smoking was more prevalent in Group 2 (33/144, 23%) than in Group 1 (41/581, 7%). Ultrasound-indicated cerclage was inserted in 14% (98/725) and preterm birth <37 weeks occurred in 10% (70/725), 24 of these despite insertion of cerclage (24/70, 34%). Fourteen delivered at <34 weeks (14/725, 2%), of which 6 (6/14, 43%) had cerclage. The background rate of preterm birth in 2005 (the midpoint of this study) across the three study units was 13% (<37 weeks) and 3% (<34 weeks). The rates in England and Wales were 11% (<37 weeks) and 3% (<34 weeks) over the same year (Table 2).

**Table 1:** Patient characteristics for women that delivered at term without cerclage (Group 1) and women that had cerclage or delivered prematurely (Group 2).

	Term birth(without intervention)Group 1, N = 581	PTB <37w or cerclage insertion Group 2, N = 144	Total population N = 725
**Age, years. Mean** (±SD, range)	33.8 (±4.2, 24–49)	33.7 (±3.6, 26–44)	33.8 (±4.1, 24–49)
**BMI. Mean** (±SD, range)	24.4 (±4.1, 18–40)	23.5 (±3.5, 18–34)	24.1 (±3.9, 18–40)
**Ethnicity,** n/N (%)			
Caucasian	381/581 (66%)	98/144 (68%)	479/725 (66%)
Asian	96/581(16%)	20 /144 (14%)	116/725 (16%)
Black	104/581 (18%)	26 /144 (18%)	130/725 (18%)
**Parity**, n/N (%)			
0	422 /581 (76%)	124 /144 (86%)	566/725 (78%)
≥ 1	139/581 (24%)	20 /144 (14%)	160/725 (22%)
**Smoker,** n/N (%)	41/581 (7%)	33 /144 (23%)*	87/725 (12%)
**Preterm birth,** n/N (%)	N/A	70/144 (49%)	70/725 (9.7%)
**Cervical Cerclage for CL <25mm, n/N (%)**			
Cerclage inserted	N/A	98/144 (68%)	98/725 (13.5%)
Preterm birth, with cerclage	N/A	24/98 (24%)	24/725 (3%)
Term birth, with cerclage	N/A	74/98 (76%)	74/725 (10%)
**CL (mm) at timepoints**, Mean (±SD) [n]			
A: 13+0–15+6	34 (±4.2) [481]	28 (±6.3)* [129]	32 (±5.3) [610]
B: 16+0–18+6	33 (±4.4) [493]	27 (±6.7)* [102]	32 (±5.4) [595]
C: 20+0–22+6	32 (±4.4) [492]	25 (±7.3)* [62]	31 (±5.2) [554]
**% ΔCL between timepoints**, Mean (±SD) [n]			
A-B	3% (±8) [426]	11% (±13)* [96]	4% (±10) [522]
B-C	2% (±9) [452]	18% (±20)* [55]	4% (±12) [507]
A-C	5% (±11) [413]	24% (±20)* [52]	7% (±14) [465]

Group 1 = delivery >37weeks without intervention; Group 2 = preterm birth <37weeks and/or cerclage. BMI = body mass index; CL = cervical length (mm); % ΔCL = percentage change in CL (mm) between screening time points; GA = gestational age; PTB = preterm birth <37 weeks; Screening timepoints: A: 13+0–15+6 weeks, B: 16+0–18+6 weeks, C: 20+0–22+6 weeks; SD = standard deviation; W = weeks

*P <0.05 for comparisons Group 1 vs Group 2

**Table 2:** Preterm birth rates: A in the UK; B in the local population (three study units); C estimated in women post-cervical treatment based on UK rates; D in this study cohort.

GA at birth	A. UK (2005)	B. Local population	C. Post-treatment estimate*	D. Our cohort
**<37 w**	11%	13%	22%	9%
**<34 w**	3%	3%	6%	2%

GA = gestational age; w = weeks

*based on Relative Risk (RR) reported in Kyrgiou Lancet 2006; Arbyn BMJ 2008

For women who received a cerclage, the mean CL and gestational age at cerclage insertion was 20mm (SD = 4.0) and 18+6 weeks (SD = 3.9) respectively. This did not differ significantly across hospital sites. Eight women (1%) who reached the CL threshold declined cerclage. Two of these delivered prematurely (<37 weeks).

**Fig 1:**
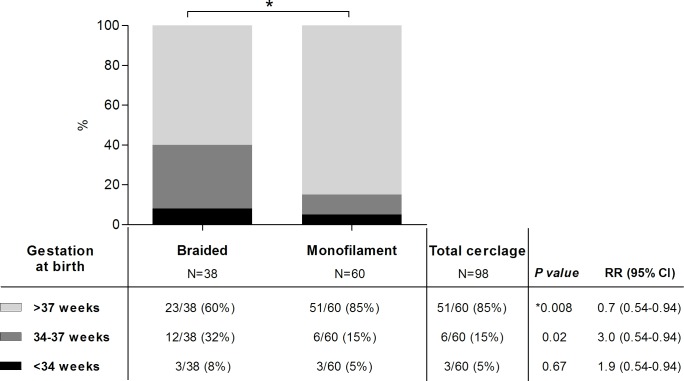
Gestation at delivery in women with an ultrasound-indicated cerclage for CL <25mm before 24weeks: a comparison of suture material braided versus monofilament. Preterm birth <37weeks was significantly higher (P = 0.08) in women with braided cerclages, compared to monofilament cerclages. This is difference is most notable among those delivering late preterm birth (34-37weeks).

Monofilament suture material was inserted in 61% (60/98) of cerclages, and the remaining received braided suture (39%, 38/98). Choice of suture material was entirely at the discretion of the operator. Although the mean CL and gestation at insertion was similar for the two suture groups, the rate of preterm birth (<37 weeks) was lower for monofilament (15%, 9/60) compared to Braided cerclages (40%, 15/38) (RR 0.7, 95% CI 0.54 to 0.94, *P* = 0.008) (Fig 1). Respective mean gestational ages at birth were 38.4 weeks (±2.8, range 27 to 42 weeks) and 37.3 weeks (±3.4, range 25 to 42 weeks; *P* = 0.056) for monofilament and braided groups. Neonatal outcome was comparably worse for those receiving braided suture material than monofilament, who had lower mean birthweights (2890 vs. 3173 grams, *P* = 0.1), lower Apgar scores at 10 minutes of age (8 vs.10, *P* = 0.03), and higher rates of admission to neonatal intensive care (5/38, 13% vs. 5/60, 8%, *P* = 0.05; Table 3).

**Table 3:** Neonatal outcome as a function of cerclage suture material.

	Braided, n = 38 Mean ±SD (range)	Monofilament, n = 60 Mean ±SD (range)	No cervical shortening/ no cerclage, n = 627 Mean ±SD (range)
**Gestation at birth (w)**	37.3 ±3.4 (25–42)	38.4 ±2.8 (27–42)	39.1 ±1.8(29–42)
**Birth weight (g)**	2890 ±873 (621–4210)	3173 ±692 (1260–4340)	3348 ±566 (1450–5074)
**Apgars**			
**1 minute**	8 ±2.3 (2–10)	8 ±1.5 (3–10)	9 ±1.3 (1–10)
**10 minute**	9 ±1.7 (5–10)	10 ±0.9* (4–10)	10 ±0.6 (7–10)
**Admission to NICU, n/N,%**	5/38, 13%	5/60, 8%	8/627, 1.3%

SD = standard deviation w = weeks gestation, g = grams, NICU = neonatal intensive care unit

*P = 0.03 t-test; Braided v monofilament

### Difference in serial CL measurements (ΔCL)

**Fig 2:**
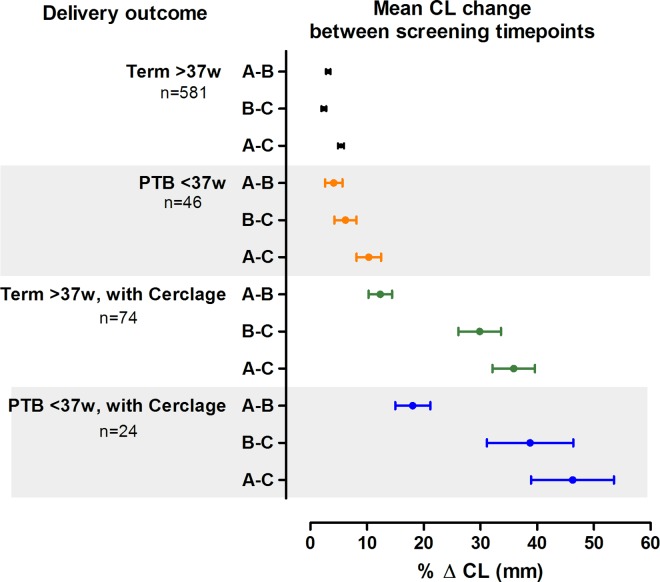
**Mean difference in CL (mean % Δ CL) between time-points A: 13+0–15+6 weeks, B: 16+0–18+6 weeks, C: 20+0–22+6 weeks (A-B, B-C, and A-C) according to delivery outcome and cerclage insertion.** In women receiving a cerclage, mean CL started above 25mm at timepoint A, and went on to shorten, most significantly at timepoint C. The greatest difference in CL is observed between timepoints B-C and A-C in those that received a cerclage and went on to deliver preterm <37weeks, followed by term delivery with a cerclage. (*% ΔCL = percentage change in CL (mm) between screening time points; PTB = preterm birth <37 weeks; Screening time points = A*: *13+0–15+6 weeks*, *B*: *16+0–18+6 weeks*, *C*: *20+0–22+6 weeks; SD = standard deviation; Term = birth >37 weeks; W = weeks)*.

For women who underwent cerclage, there was no difference in the mean CL of those delivering before or after 37 weeks (*P* = 0.4; Table 4), indicating that the CL at insertion of cerclage did not predict preterm birth post-cerclage. The largest reduction in CL (%ΔCL) was observed in those requiring a cerclage, regardless of eventual gestation at birth (*P*<0.05) (subgroups of Group 2) (Fig 2, Table 4). The difference in CL was greatest for comparisons made screening after 20 weeks (timepoint C: 20^+0^–22^+6^ weeks), indicating that these women at high-risk start with a reassuring CL before 20 weeks (above 25mm), and go onto shorten (Fig 2, Table 4). The sensitivity of single CL measurements in predicting preterm birth improves with advancing screening gestation (from A to C) and increasing CL thresholds (Table 5).

**Table 4:** Mean CL (mm)(SD) at screening time-points A, B, C and mean percentage ΔCL (SD) between screening time-points A-B, B-C, and A-C for Group 1 and 2.

Screening timepoints (w)	Group 1 Birth >37weeks without cerclage N = 581	Group 2 PTB <37weeks and/or cerclage N = 144	Group 2 subgroups			Total scanned, N = 725
			PTB (no cerclage) N = 46	PTB with cerclage N = 24	Term with cerclage N = 74	
	Mean CL (mm) (SD) [n]					
**A: 13+0–15+6**	33.6 (4.2) [481]	28.0* (6.3) [129]	32.3 (6.0) [39]	26.8* (5.4) [22]	25.8* (5.4) [68]	610
**B: 16+0–18+4**	32.8 (4.4) [493]	26.8* (6.7) [102]	32.3 (6.2) [35]	24.5* (5.2) [15]	23.8* (5.2) [52]	595
**C: 20+0–22+6**	31.8 (4.4) [492]	25.1* (7.3) [62]	30.0 (4.5) [35]	16.4* (5.9) [8]	19.7* (4.4) [19]	554
**Difference between screening timepoints**	**Mean % ΔCL (SD) [n]**					
**A-B**	3% (8) [426]	11%* (13) [96]	4% (8) [31]	18%* (12) [16]	12%* (15) [49]	522
**B-C**	2% (9) [452]	18%* (20) [55]	6% (11) [30]	39%* (23) [9]	30%* (15) [16]	507
**A-C**	5% (11) [413]	24%* (20) [52]	10% (12) [28]	46%* (21) [8]	36%* (15) [16]	465

CL = cervical length (mm); % ΔCL = percentage change in CL (mm) between screening time points; GA = gestational age; Group 1 = delivery >37weeks without intervention; Group 2 = preterm birth <37weeks and/or cerclage; ns = not significant; PTB = preterm birth <37 weeks; Screening time points: A: 13+0–15+6 weeks, B: 16+0–18+6 weeks, C: 20+0–22+6 weeks; SD = standard deviation; Term = birth >37 weeks; W = weeks

*P<0.05 for comparisons of mean CL & % ΔCL for Group 1 vs Group 2, and Group 1 vs Group 2 subgroups, according to screening timepoints A, B and C and A-B, B-C and A-C respectively.

**Table 5:** Sensitivity, specificity, likelihood ratios, and positive and negative predictive values for cerclage intervention and/or preterm birth <37 weeks, for screening time-points A, B and C, and percentage difference in CL between screening time-points A, B and C.

Screening time-points (w)	CL threshold (mm)	S (%)	Sp (%)	PPV (%)	NPV (%)	LR
**A: 13+0–15+6**	**≤ 20**	12	100	100%	81%	27.9
** **	**≤ 25**	30	99	85%	84%	20.6
** **	**≤ 30**	73	74	44%	91%	2.9
** **	**≤ 35**	94	29	26%	95%	1.3
** **	**≤ 40**	97	4.6	22%	95%	1
** **	**≤ 50**	100	0.2	21%	100%	1
**B: 16+0–18+6**	**≤ 20**	17	100	95%	85%	85.3
	**≤ 25**	41	98	84%	89%	25.5
** **	**≤ 30**	78	68	34%	94%	2.5
** **	**≤ 35**	92	24	20%	94%	1.2
** **	**≤ 40**	99	5.1	18%	96%	1
** **	**≤ 50**	100	0.2	17%	100%	1
**C: 20+0–22+6**	**≤ 20**	29	99	82%	92%	35.1
** **	**≤ 25**	51	95	57%	94%	10.4
** **	**≤ 30**	75	56	18%	95%	1.7
** **	**≤ 35**	97	21	13%	98%	1.2
** **	**≤ 37**	100	13	13%	100%	1.2
**Difference between screening time-points**	**ΔCL threshold (mm), %**	**S (%)**	**Sp (%)**	**PPV (%)**	**NPV (%)**	**LR**
**A-B**	**≥ 5%**	66	60	27%	89%	1.6
	**≥ 10%**	49	83	39%	88%	2.8
	**≥ 20%**	20	97	59%	84%	6.5
	**≥ 30%**	10	99	77%	83%	14.8
	**≥ 40%**	1	100	100%	82%	1
**B-C**	**≥ 5%**	73	62	19%	95%	1.9
	**≥ 10%**	56	84	30%	94%	3.5
	**≥ 20%**	44	98	69%	93%	17.9
	**≥ 30%**	24	100	87%	91%	53.4
	**≥ 40%**	13	100	100%	90%	57.5
**A-C**	**≥ 5%**	79	47	16%	95%	1.5
	**≥ 10%**	73	69	23%	95%	2.3
	**≥ 20%**	48	91	40%	93%	5.4
	**≥ 30%**	37	98	70%	92%	18.9
	**≥ 40%**	19	99	77%	91%	26.5

CL = cervical length (mm); % ΔCL = percentage change in CL (mm) between screening time points; LR = Likelihood ratio; NPV = negative predictive value; PPV = positive predictive value; S = sensitivity; Screening time points: A: 13+0–15+6 weeks, B: 16+0–18+6 weeks, C: 20+0–22+6 weeks; Sp = specificity; W = weeks.

A 100% NPV for preterm birth >37 weeks is associated with CL >50mm, >50mm, and >37mm at screening timepoints A, B and C respectively. Observation of CL reduction before 20 weeks was highly specific for prediction of preterm birth <37 weeks and/or cerclage (99% specificity when ΔCL ≥30% at timepoints A-B; Table 5), although sensitivity remained poor. The optimum balance between sensitivity and specificity was a ΔCL ≥10% at A-C, associated with high negative prediction (95%).

### Triage Screening Model

**Fig 3:**
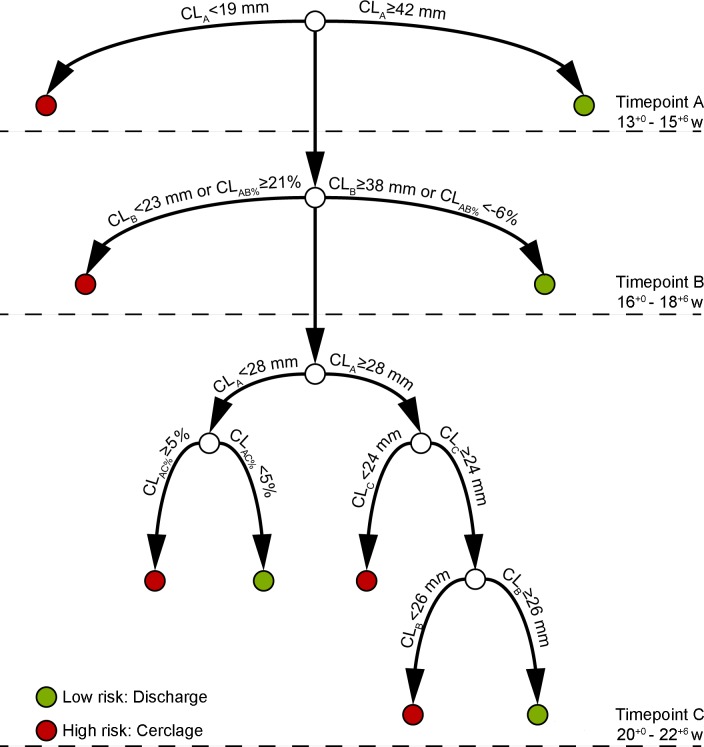
Triage Screening Model for pregnancies post excisional cervical treatment for the prevention of preterm birth <37weeks. A triage screening model was developed using decision tree analyses to determine optimum thresholds of CL and % change in CL between screening timepoints A (13+0–15+6 weeks), B (16+0–18+6 weeks) and C (20+0–22+6 weeks), to ensure appropriate allocation of resources. This model identifies pregnancies at low-risk of preterm birth enabling safe and timely discharged from cervical length surveillance (green dot). Similarly early identification of high-risk pregnancies allows for timely cerclage intervention (red dot). Serial CL surveillance can therefore reserved for pregnancies considered at intermediate risk, requiring further observation. *CL = cervical length (mm); CL*_*AB%*_ *= percentage change in CL (mm) between screening time points; w = weeks*.

Based on this analysis, we developed a Triage Prediction Model using discriminatory analyses to identify women with a history of cervical treatment at low risk of preterm birth <37 weeks and/or cerclage and applied this in our cohort (Fig 3). At timepoint A (13^+0^–15^+6^ weeks), a CL threshold of <19mm identified 13% of the women who went onto deliver preterm (9/70). At the same screening timepoint, 40 of the 581 low risk women (7%) were identified using a CL threshold ≥42mm. Of the remaining 676 women with CL between ≥19mm and <42mm, 24% (135/144) went on to require either a cerclage or deliver preterm, however these were not distinguishable from the remaining 541 low risk women. This group were considered at intermediate risk and required subsequent screening at timepoint B (16^+0^–18^+6^ weeks). At timepoint B, a CL <23mm or a reduction in CL of ≥21% from timepoint A identified a further 38/144 (26%) high risk women going onto deliver preterm or receive a cerclage, and a CL ≥38mm or a reduction in CL of <6% at the same screening timepoint identified 89 (15%) low risk women. The remaining 552 women were considered at intermediate risk, of which 100 (18%) would require either a cerclage or would go onto deliver preterm. This intermediate risk group then went on to require screening at timepoint C (20^+0^–22^+6^ weeks), where a further discriminatory analysis was performed based on initial CL at timepoint A. CL <28mm at timepoint A with ≥5% reduction in CL or a CL ≤24mm at timepoint C, identified 38 and 16 women at risk of preterm birth respectively. A CL ≥24mm at timepoint A, but <26mm at timepoint B identified 46 women at risk of preterm birth, while the remaining 452 women were low risk.

Assuming all patients attended initial screening at timepoint A and attended for at least three TVUS, we computed that 23% of follow up scans would have been unnecessary. More specifically, 6% (40/725) of women would have been discharged immediately after initial screening, and further 22% (156/725) would require only one additional scan. This would equate to a substantial reduction in unnecessary follow up scans (236/1450). Furthermore, after discharge of low-risk women and cerclage insertion for high-risk women, only 36% of the population (n = 258/725) would have benefitted from screening at all three timepoints; application of this model would ensure focused allocation of resources to this specific group most likely to shorten with the highest risk of preterm birth.

## Discussion

The antenatal management of women with a previous cervical treatment for CIN varies, while evidence on how to best manage this population is lacking. Many obstetricians believe that preterm birth following cervical excision is a result of ‘cervical weakness’, which can be corrected by cerclage. Evidence thus far indicates that this may not be the case and therefore the risk of preterm labour could plausibly be unaffected or even worsened by cervical cerclage[17–20, 32]. As these reports were exclusively in cohorts in which braided suture was used for cerclage, we hypothesized the reported lack of cerclage efficacy in pregnancy post-conization, relates to the effects of ‘foreign’ material (cerclage) on the vaginal microenvironment[23–25] and the immune system[33]. Braided suture is the current material of choice for the cervical cerclage despite a lack of evidence to support its preferential use [17, 21, 22]. This is the first study to reveal an advantage of using a monofilament suture in pregnancies post-conization for CIN with a shortened cervix to <25mm. Furthermore, with the proviso that the cerclage is of monofilament suture, ours is the first study to indicate that the policy of targeted cervical cerclage is beneficial in preterm birth prevention. In our cohort of women post-conization the rate of preterm birth was below the baseline rate for the general obstetric population across the three study hospitals sites and below the overall rates for England and Wales. We estimated the expected rates of preterm birth in our cohort without specific management as a 1.5–2 fold increase [2, 5, 34]. Our management protocol therefore reduces the risk of preterm birth in pregnancy post-conization to a rate similar to that of the general background obstetric population.

Measurement of serial second trimester CL is increasing used for surveillance of women at risk of preterm birth to balance the high specificity of early screening (<16weeks), with improved sensitivity at later screening (>22weeks) [1, 10, 11]. Observation of the rate of cervical change between screening is also predictive of preterm birth in general obstetric populations [35–40]. The only study assessing cervical change in women post-LLETZ concluded that it does not predict preterm birth [41]. Although this appears to contradict our findings, this may explained by that study’s design. All women with cervical shortening deemed sufficient to warrant an ultrasound indicated cerclage (the highest risk of preterm birth) were excluded, while a 17% rate of preterm birth in the remaining population questions the quality and reliability of the screening program. We excluded from our cohort women with additional risk factors for preterm birth (previous preterm deliveries, uterine anomalies, multiple pregnancies etc.) to ensure that our screening algorithm applied specifically to those whose only risk was prior cervical conisation.

This study finds that in pregnancy post-conization the observation of change in second trimester cervical length is most valuable in the management of women whose CL is between 25mm and 30mm at screening. This challenging group makes up a significant proportion of the workload in our prematurity clinic; the positive predictive values of a single CL measuring between 25mm and 30mm do not warrant the risks of cerclage, yet the negative predictive values are not reassuring enough to discharge women from surveillance. The observation of percentage change in CL between screening time-points in these women provides clinically relevant information. A small (eg <10%) or large (eg >30%) reduction in length between screening time points justifies either a timely discharge or intervention respectively. Furthermore, this study provides optimal gestational ages for screening for both serial and single CL measurements to achieve high positive and negative predictive values for preterm birth. The Triage Prediction Model demonstrates that if CL measurement screening begins before 16 weeks, only 36% of women will require screening at all three second trimester timepoints. It ensures timely identification of women at low risk who may be safely discharged, thereby focusing allocation of resources to the women at highest risk of preterm birth. The Triage Prediction Model provides clinicians with a user-friendly, cost-effective tool for preterm birth prevention in pregnancies following excisional cervical treatment.

While we propose this model of management, its efficacy and cost effectiveness needs to be prospectively assessed in further observational studies before we can comment on the true validity of the model’s application in the general obstetric population. Preventative strategies other than the cerclage, including the cervical pessary and progesterone have not yet been evaluated in this discrete clinical group with cervical damage [13, 42, 43].

The strength of this study is that describes the largest multi-centre cohort of a homogenous population of women after cervical treatment attending for intensive antenatal surveillance in specialist prematurity clinics over a 10-year study period. The longitudinal construct of the study ensures that the sequential assessment of cervical change is from a single person and reduces potential error from individual participant variability.

The limitations of the study relate to its retrospective design and the lack of a direct comparison population (women with prior excisional cervical treatment that did not attend screening). This was an important source of bias, particularly in the earlier years of the study, when the association between conization and preterm birth was not well-established, and preterm clinic referrals were unlikely to reflect all women post-conization within the population. Another source of bias was a lack of randomization of suture material at cerclage insertion. Operators may have been biased towards one suture material depending their own pre-conception as to superior suture material and how high risk they perceived the participant. This bias was considered minimal as all operators appeared to use only one suture material throughout the study period. A further limitation was a lack of available data with respect to specific neonatal outcome including oxygen supply, mechanical ventilation, neonatal sepsis, and neonatal brain lesions such as leucomalacia or interventricular haemorrhage.

Although the Shirodkhar and MacDonald cerclage techniques have been shown to be equally efficacious [44], we were also unable to incorporate cerclage techniques into our analysis as this was not always clearly specified in all operation notes; another limitation of retrospective data collection. Due to an ethical obligation to intervene in women with a shortened cervix in pregnancy, a control group with a short cervix but without a cerclage in which to compare preterm birth rates, does not exist in current clinical practice. This is an unavoidable limitation of clinical preterm birth research.

## Conclusion

In pregnancy following excisional cervical treatment for CIN, insertion of a non-absorbable monofilament cerclage for a shortened cervix effectively reduces the preterm birth risk and is preferable to a braided cerclage. Using our Triage Prediction Model pregnancies at low-risk of preterm birth may be identified and discharged from preterm cervical length surveillance in a cost effective and timely manner.
